# Masculinities and violence: using latent class analysis to investigate the origins and correlates of differences between men in the cross-sectional UN Multi-country Study on men and violence in Asia and the Pacific

**DOI:** 10.7189/jogh.10.020439

**Published:** 2020-12

**Authors:** Rachel Jewkes, Esme Jordaan, Henri Myrttinen, Andrew Gibbs

**Affiliations:** 1 Gender & Health Research Unit, South African Medical Research Council, Pretoria, South Africa; 2 Office of the Executive Scientist, South African Medical Research Council, Pretoria, South Africa; 3 School of Public Health, Faculty of Health Sciences, University of the Witwatersrand, Johannesburg, South Africa; 4 Biostatistics Unit, South African Medical Research Council, Cape Town, South Africa; 5 Gender Associations International Consulting, Berlin, Germany; 6 Centre for Rural Health, School of Nursing and Public Health, University of KwaZulu-Natal, Durban, South Africa

## Abstract

**Background:**

Multiple masculinities have been explicated through latent class analysis (LCA) in South Africa, and a question arises as to whether men can be similarly grouped by their behaviour in very different cultural contexts, and whether an analysis would point to similar origins to men’s use of violence against women. The UN Multi-country Study on Men and Violence in Asia and the Pacific’s data set enabled this question to be explored.

**Methods:**

In nine sites in six countries, data were collected from one man (18-49 years) interviewed in each of a random sample of households. Using LCA, we categorised men based on their probability of having engaged in 10 acts of violence against women or other illegal or sexually risky behaviour. We present multinomial logistic regression models of factors associated with class membership and associated childhood and trauma experiences.

**Results:**

The LCA model with 5 classes fitted best: the largest class (59.5% of men) had the lowest probabilities of engagement in the class-defining acts; men in the second (21.2%) were otherwise law abiding and not sexually risky, but very violent towards partners; men in the third (7.9%) had the highest probability of engagement in all violent and illegal behaviour; men in the fourth (7.8%) demonstrated behaviour at the nexus of sex and power including rape and transacted sex; and men in the fifth (3.6%), engaged in anti-social behaviour, but were less violent towards women and sexually risky. Assignment to more violent classes was associated with poverty, substance abuse and depression, and more gender inequitable attitudes and practices. Child abuse, neglect and bullying were associated with being in the more violent classes. Neither men’s domestic practices nor their fathers’ presence in their childhood were associated with class.

**Conclusions:**

Closely paralleling the South African findings, we have highlighted the childhood origins of men’s violent and anti-social behaviour, as well as the interrelationships with men’s mental health, poverty and misogyny, showing that these (intersectional) developmental processes transcend culture and setting. We need to prevent children’s exposure to violence, and in gender transformative work with men, recognise and address past and present psychological distress stemming from trauma experience.

Men’s perpetration of violence against women, violence against other men and sexually risky practices track together [1-4]. Gender activists and scholars have argued that this stems from particular constructions of masculinity that arise within the global system of patriarchy, structurally positioning men as superior to women. Masculinities are the ways of being a man, and these connect with and give expression to social values, roles, attributes, behaviours and aspirations [5-7]. Much of the work describing the interconnections comes from studies of African men’s HIV-related risk behaviours and gender inequitable practices [8-10], and the extent to which this pertains in other global regions, has been less explored. However in many global settings, the practices of men’s violence against women, violent and anti-social behaviour involving other men, and behaviour at the nexus of sex and power have been described [11-14]. A key question relates to whether such associations stem from local culture, or whether they arise from a broader configuration of masculinities within the global system. The data set of the UN Multi-country Study on Men and Violence provides an opportunity for investigating this as it is drawn from nine very diverse settings within Asia and the Pacific.

The theoretical basis for this analysis is the argument that men’s behaviour stem from expressions of masculinity and men’s differing positions in respect of the exercise of their male power and perceived entitlement, particularly, but not exclusively, over women [14,15]. Connell’s theorizing of masculinity, including the concept of hegemonic masculinity, elaborated by other authors, provides a framework for understanding how different forms of masculinity are found in any setting and express different ways in which men view themselves as men, ie, their aspirations, attitudes and expectations, conveyed through their practices [6,16-18]. In a context underpinned by patriarchal power, these practices are importantly performed through gender relations with women. The gender hierarchy is maintained, and sexual access to women secured, through a spectrum of means from freely given consent or normative acquiescence to the use of manipulation, threats and violence and payment for sexual services. Masculinities also centrally relate to men’s hierarchy with respect to other men, both through hierarchical power of different masculinities elaborated by Connell, and competition at an individual or group level among men [7,19,20]. Masculinities are not all expressed in ways that are toxic for women, but also include among them ways of being men that are more equitable, less violent and often include men who actively resist gender inequitable and violent masculinities of other men [15,17,21].

Whilst much of the research elaborating different constructions of masculinities has been qualitative, there have been quantitative approaches to operationalize the idea using the approach of Latent Class Analysis (LCA). Using data from a population-based survey in South Africa, Jewkes and Morrell used this technique to identify an underlying structure of three classes of men based on their probability of having engaged in a range of acts of violence against women and other men, and other sexually exploitative and anti-social behaviour [15]. The largest class was the least violent, although not a majority class, and the authors proposed that the other two classes represented a hegemonic masculinity in that setting with the most violent class expressing the hypermasculine exaggeration of the hegemonic [21]. Other samples from South Africa have shown similar findings, but the relative size of groups varied. Just under a third (29.4%) of young men (18-30) were allocated to the most violent group in urban informal settlements in KwaZulu Natal Province, compared to 15.5%, in an older community-based sample from similar areas in Gauteng Province [22,23].

This work has been important in understanding the quantitative aspects of the configuration of masculinities within settings, as well as across age and local context. It has provided a different dimension to understanding the origins of different masculinities, and has opened up a range of research questions related to efforts to change masculinities, notably the question of which groups of men are impacted by interventions [23,24]. Our analysis has three aims. The first is to identify different masculinities using LCA, based on the clustering of practices, and compare the prevalence of these classes across different settings in Asia-Pacific. The second aim is to describe the social and attitudinal factors independently associated with the masculinities classes across the six settings/countries of Asia-Pacific. We hypothesise that as in South Africa, men’s social and demographic characteristics, childhood experiences, psychological factors and substance abuse and gender attitudes and division of work are associated with masculinity class. The third aim is to describe the association between the different classes and childhood experiences, which we hypotheses precede the adoption of particular masculine identities.

## METHODS

We conducted a secondary analysis of data from the UN Multi-country Study of Men and Violence in Asia and the Pacific, which was developed by Partners for Prevention, a United Nations Development Programme (UNDP), United National Population Fund (UNFPA), UN Women and United National Volunteers (UNV) regional joint programme for gender-based violence prevention in Asia and the Pacific, in collaboration with the Medical Research Council of South Africa and the country research teams. The first author was lead technical adviser for this study and Principal Investigator in Bougainville. Field research was conducted in 2011-12 in nine diverse sites in six countries in Asia and the Pacific: Bangladesh, China, Cambodia, Sri Lanka, Indonesia and Papua New Guinea (Bougainville). The sample was nationally representative of Cambodia and Bougainville. In Bangladesh and Indonesia there was a site in the capital city and one or two provincial sites, respectively. Of the Indonesian provincial sites, one was a rural site (Java) and the other a mostly urban site with peri-urban and rural settlements (Papua). The Chinese site was a county with a town and rural area, and in Sri Lanka, Colombo and three contrasting districts were surveyed. Full details on the study methods, sampling, response rates, questionnaires and the findings on intimate partner violence (IPV) in these same study sites are presented elsewhere[25]. The sites differ greatly. They were culturally and religiously diverse, and differed in their socio-economic profile, housing conditions, political and educational systems, access to services and information, urbanity, family/kinship structures and in their history of civil conflict. All of these factors influenced expectations of what it means ‘to be a man’ in a given society and the possibilities of achieving these expectations, but also various stress factors that may impact on IPV perpetration.

A two-stage sampling strategy was used to identify census enumeration areas, with a probability proportionate to size, and randomly select households within these areas. In each household a man aged 18-49 years (where necessary, randomly selected) was invited for interview, with a trained male interviewer. The sample was mostly of heterosexual men, but this was not an inclusion requirement. Most interviews were face to face, but answers to most sensitive questions were provided by self-completion on Audio-enhanced Personal Digital Assistants (APDAs). In China a household list of individuals in each cluster by age and sex was available and so used for sampling within selected clusters and the entire questionnaire was self-completed. 10 178 number of men were interviewed, between 799–1776 per site. The proportion of enumerated and eligible men interviewed per site varied between 59% and 95%, and only in urban Bangladesh and Sri Lanka was it below 80%[25]. The variables used in the analysis are presented in **Table 1**.

**Table 1:** Operational definitions of variables

Explanatory variables	
**Social characteristics**	**Definition**
No high school	Only attended primary school education or had no education.
Current food insecurity	Sometimes or often people at home go without food because of a lack of money.
**Victimization history and childhood**	
Paternal absence	When growing up, biological father rarely or never at home.
Father’s contribution to housework	When growing up father or step-father prepared food, cleaned the house, washed clothes or cared for the respondent or his siblings (4 items scored 1-4 never, sometimes, often and very often). Summed and a categorical dummy variable derived: 1 = score <8, 2 = 8-11, 3 = 12-16.
Childhood emotional abuse or neglect (often)	Men were asked about their experiences of trauma in childhood using a modified version of the Childhood Trauma Questionnaire. Before age 18 y respondent had at least one of the following experiences sometimes, often or very often: lived in different households at different times; was told he was lazy or stupid or weak by someone in his family; was insulted or humiliated by someone in his family in front of other people; both of his parents were too drunk or drugged to take care of him; witnessed his mother beaten by a partner; spent time outside the home and none of the adults at home knew where he was.
Childhood physical abuse	Before age 18, respondent had at least one of the following experiences sometimes, often or very often: was beaten at home with a belt or stick or whip or something else which was hard; was beaten so hard at home that it left a mark or bruise.
Childhood sexual abuse	Before age 18, respondent had at least one of the following experiences sometimes, often or very often: someone touched his buttocks or genitals or made him touch them when he did not want to; had sex with someone because he was threatened or frightened or forced.
Witnessed abuse of his mother	Before age 18, the respondent saw or heard his mother being beaten by her husband or a boyfriend.
Teased or bullied as a child	Respondent was bullied, teased or harassed in school or in the neighbourhood in which he grew up.
Experienced homophobic violence or teasing	Had been called names, faced derogatory remarks or been subjected to violence or threats because they were thought to be effeminate or attracted to men. 2 items scored and a dummy variable derived with 0 = never 1 = ever experienced this.
**Gender attitudes and relationship practices**	
Low gender equity	Tertiles created from 10 items scored on a 4-point scale from strongly agree to strongly disagree and this variable refers to the lowest tertile − Typical item “A woman’s most important role is to take care of her home and cook for her family”. Alpha = 0.72
Respondent dominates some or all decisions at home	4 items asking about health of women, decisions around children (if any), spending money on food and clothing and large investments. Each question asked about who made the decisions: himself, his wife/partner, both equally or another person in the household. Derived variable captured men who himself made some or all of these decisions vs other response options.
Relationship control	8 items with Likert responses: strongly agree, agree, disagree, strongly disagree, summed. Typical item “When I want sex I expect my partner to agree”. Dummy variable created with categories cut at tertiles of the distribution, with a category derived for missing values, including men without a partner.
Ever sex with sex worker	Respondent has ever had sex with a male, female or transgender sex worker.
Transactional sex ever	4 items: Ever had sex with a woman/girl in exchange for drugs, food, cosmetics, clothes, a cell phone, transportation; somewhere to stay; something for her children or family; or money to pay her bills or school fees. Binary variable derived with ever v. never categories.
>1 sexual partners in the past year	Number of different people respondent has had sex with in the past year including his main partner, multiple response categories. Binary variable created for 0/1 v. 2 or more.
**Psychological factors and substance abuse**	
Depression	20 item CES-D depression score, cut point of 16+ taken as indicating a high level of depressive symptomatology.
Life satisfaction	Measure of satisfaction with current life circumstances based on 4-items scored on a 4-point scale from strongly agree to strongly disagree – In most ways my life is close to my ideal; The conditions in my life are excellent; I am satisfied with my life; So far I have gotten the important things I want in life. Alpha = 0.79.
Alcohol problems	Based on AUDIT scale: frequency of drinking, number of drinks usually consumed, frequency of binge drinking (6+ drinks) and feelings of guilt or remorse after drinking and failure to do what was normally expected from you because of drinking
**Engagement in violence outside the home and drug use**	
Involvement in gangs	Has ever participated in a gang.
Involved in fights with weapons	Has ever been involved in a fight with a knife, gun or other weapon.
Past year drug use	Has ever used drugs in the last 12 months.
Violence against women:	
Non-partner rape ever	2 items asked about having ‘forced a woman who was not your wife or girlfriend at the time to have sex’ or having ‘had sex with a woman who was too drunk or drugged to indicate whether she wanted it’. Two more asked about having done these with other men. A binary variable was derived from responses categories as ever v. never.
>1 episode of physical and/or sexual IPV	5 items on physical intimate partner violence perpetration were South African adaptations for men from the WHO Multi-country Study measure. 2 items on sexual violence against a partner asking about ‘forced sex’ or sex ‘forced when he knew she didn’t want it but believed she should agree because she was his wife/partner’ (this second question was not included in Bangladesh). Each had never, once, few, many response options. Variable was derived categorizing responses into never or one act of physical/sexual IPV vs more than one.
>1 episodes or acts of emotional IPV	5 items from the WHO Multi-country Study measure asked about ever: insulting his partner, scaring her, threatening her, humiliating her, damaging property or people who cares about. Each had never, once, few, many response options. Variable was derived categorizing responses into never or one act vs more than one.
>1 episodes or acts of economic IPV	4 items from the WHO Multi-country Study measure asked about ever: Prohibited a partner from getting a job, going to work, trading or earning money; took her earnings against her will; threw her out of the house; kept money from his earnings for alcohol, tobacco or other things for himself when he knew his partner was finding it hard to afford the household expenses. Each had never, once, few, many response options. Variable was derived categorizing responses into never or one act vs more than one.

### Statistical analysis

For this sample of men, latent class analysis was used to identify latent unobserved subgroups, comprised of similar individuals, using the relationships among a set of responses to the categorical questions (indicators) on aspects of men’s practices: (1)>1 episode ever of physical violence, sexual IPV or both, (2) ever been on a fight with a weapon, (3) ever participated in a gang, (4) any past drug use, (5) any transactional sex ever, (6) sex with sex worker, (7)>1 acts of emotional abuse ever, (8)>1 acts of economic abuse ever, (9)>1 partner in the past year, (10) rape of non-partner ever. In preparing the variables for the latent class analysis, never partnered men were classed as ‘never’ having done an act of IPV. The class membership probabilities identified men belonging to the same class. The probability attached to each individual accounts for the fact that the classification is uncertain. The conditional probabilities, (item-response probabilities), the probability of endorsing each indicator within a class, provides the class description. We have allocated a description depending on the set of indicators that are endorsed with a high probability for each class: thus if question 1 and 2 were endorsed with a very high probability the class could be named “violent class”. Various model fit indices were used to decide on the number of classes to obtain the optimal model (see below). An item-response probability of 0.5 indicates that half of the group members said “yes” and the other half said “no” to the specific item. In labeling the classes we have considered item-response probabilities larger than 0.5.

Each of the men were classified into a specific class according to their highest probability. We present descriptive statistics (percentage of men and means) for different independent variables by class test the association for categorical variables with a Pearson chi squared test. Throughout the analysis Taylor linearization was used to take into account the clustering of men into primary sampling units and the stratification of the data by country/site. The latent class nominal variable was then used in further multinomial logistic regression analysis as an outcome variable. The variables were divided into childhood variables and indicators of the current social circumstances, psychological state and gender attitudes and practices of men. A model was built containing all the current state indicators including a variable for stratum (country/site). Variables were not eliminated based on their *P* value, but we use a *P* value of less than 0.01 for interpreting the multiple logistic regression model. This model only included 9753 partnered men with complete data, dummy categories were created for the relationship control and male dominance of decision-making variables to prevent loss of cases from the model due to men not being partnered. A second model was built in a similar manner, with the latent classes as the outcome, but with the childhood variables. Again, there was no elimination of non-significant variables. In total 8058 men had complete data and were included in this model.

### Ethics

We followed ethical and safety guidelines for research with men on perpetration [31]. To protect men providing sensitive disclosure, we presented the study as a family and health study. The interviewees received an information sheet and provided signed informed consent. We kept no household lists with identifying details of respondents. Ethical approval was provided by the South African Medical Research Council; the College of Humanities, Beijing Forestry University; International Centre for Diarrhoeal Disease Research, Bangladesh; National Ethics Committee for Health Research of Cambodia; the Faculty of Medicine at the University of Colombo, Sri Lanka; and Faculty of Medicine, Gadjah Mada Univerity, Indonesia.

## RESULTS

The latent class model with the best fit was obtained with 5 classes. The table with the various model fit indices comparing the models are provided in Table S1 in the **Online Supplementary Document**. Table 2 shows the five classes and the proportion of men in the data set in each class. Class 5 was the largest comprising 59.5% of men and this was the class in which men had the lowest conditional probabilities of having engaged in various acts of violence against women, illegal and other violent behavior and multiple sexual partnering. The second largest class (21.2%) was class 2 (violent against partners, but otherwise law abiding), and this was the class where, like class 5, there was a small probability for men to engage in illegal acts and other violence, including having sex with a sex worker, and few men had had multiple sexual partners, but the probabilities of acts of violence against women partners- physical, sexual, economic and emotional, and relatedly transactional sex -were higher.

**Table 2:** Distribution of variables by violent masculinity category (all countries)

	Class				
	**Very violent, risky and anti-social**	**Law abiding but violent against women partners**	**Nexus of sex and power**	**Community violence**	**Prosocial and least violent**
	**1**	**2**	**3**	**4**	**5**
	**Class membership probabilities**				
	801 (7.9%)	2156 (21.25)	798 (7.8%)	371 (3.6%)	6051 (59.5%)
	**Conditional probabilities for each class**				
>1 episode of physical and/or sexual IPV	0.873	0.560	0.279	0.163	0.078
Ever been in a fight with a weapon	0.535	0.081	0.080	0.696	0.029
Gang member	0.497	0.036	0.045	0.553	0.015
Past year drug use	0.308	0.051	0.108	0.352	0.056
Any transactional sex ever	0.677	0.257	0.640	0.287	0.182
Sex with a sex worker:	0.537	0.067	0.589	0.181	0.020
>1 episodes or acts of emotional IPV	0.863	0.881	0.337	0.374	0.157
>1 episodes or acts of economic IPV	0.793	0.536	0.338	0.323	0.161
>1 past year sexual partners	0.620	0.123	0.487	0.287	0.053
Ever raped a woman non-partner	0.708	0.077	0.223	0.142	0.001

IPV – intimate partner violence

The third most common class was class one (7.9%) (violent, sex seeking and illegal behaviours), which was the class where the men had the highest probability of engaging in all forms of violence and illegal behavior (except any past year drug use, which was slightly higher in class 4). The fourth most common group (7.8% of men) was Class 3 (nexus of sex and power). The men in this class were also not very likely to have engaged in illegal behaviours and other violence, but violence against women partners was moderately common with between a quarter and a third of men having engaged in different types. However, behaviours at the nexus of sex and power were very common. 22.3% of men in this class had raped a non-partner, 64% reported engaging in transactional sex and 58.9% had had sex with a sex worker. Finally, the smallest class was number 4 (3.6%), which was marked by high probabilities of having engaged in acts of community violence (gangs and fights with weapons).

**Table 3** shows the distribution of the classes by country and site. The pattern of class distribution was broadly similar across all countries except Bougainville, Papua New Guinea. In all other countries and sites, the most common class was the least violent and most law abiding, Class 5. In the other countries and sites between 41.2% (Indonesia Papua) and 72.4% (Rural Java) of men were in this class. In all settings except Bougainville, the second most common class was Class 2 (generally law abiding, but violent against partners) with between 12.1% (Sri Lanka) and 25.4% (Indonesia Papua) of men were in this Class. This class was the one which was most common in Bougainville (35.5% of men). There was greater variation between countries and sites in the relative sizes of Classes 1, 3 and 4. Class 1, the very violent, sex seeking and illegal behaviours class, was more common in Bougainville (30.3% of men) than in other sites. It was the third most common Class in Indonesia Papua (18% of men). A much smaller proportion of men were in this class in other settings. In contrast Class 3, emphasizing the nexus of sex and power, was the third most common in many of the settings, with 12.1% and 15.7% of men in this class in Cambodia and China respectively. There were relatively few men in the community violence class (Class 4) across settings, with only Sri Lanka standing out where it was the third most common class, with 7.2% of men.

**Table 3:** Distribution of men into masculinity categories by country and site

Class	Total	Bangladesh rural	Bangladesh urban	Cambodia	China	Sri Lanka	Papua New Guinea Bougainville	Indonesia Jakarta	Indonesia Rural Java	Indonesia Papua
**1**	801	31	32	115	65	67	262	48	20	161
%	7.9	2.7	2.6	6.4	6.5	4.4	30.3	5.5	2.5	18
**2**	2156	248	222	456	177	186	315	171	154	227
%	21.2	21.7	17.7	25.2	17.8	12.1	35.5	19.7	18.9	25.4
**3**	798	62	83	220	156	62	34	48	31	102
%	7.8	5.4	6.6	12.1	15.7	4	3.9	5.5	3.8	11.4
**4**	371	11	20	46	21	110	57	51	20	35
%	3.6	0.96	1.6	2.5	2.1	7.2	6.6	5.9	2.5	3.9
**5**	6051	791	895	975	578	1108	196	550	590	368
%	59.5	69.2	71.5	53.8	58	72.3	22.7	63.4	72.4	41.2
Total	10177	1143	1252	1812	997	1533	864	868	815	893

**Table 4** and **Table 5** show bivariate associations between class membership and variables indicative of social and demographic characteristics, childhood violence exposures, psychological variables and gender attitudes and practices. In all but two settings (Indonesia Rural Java and Indonesia Papua) there was a significant association between age and class membership. Class 4 (high on weapons, gangs and drug use, but less so on IPV) was consistently associated with the youngest age group of men. In Indonesia Papua, Cambodia and urban Bangladesh, there was a very similar proportion of men in different age groups of the least violent men (class 5) category. In other settings, apart from Bougainville, being in the least violent class (class 5) was associated with being in the oldest age group. In every setting more men of the oldest age group were in class 2 (low on other violence and illegal and sex seeking but high on IPV). In most settings men of the most violent and anti-social behavior category (class 1) were most likely to be 25-34 years, the exceptions were rural Bangladesh, Cambodia and China where they were most likely to be in the oldest age group.

**Table 4:** Prevalence of possible associated factors by masculinity class, Bangladesh, China, Cambodia and Sri Lanka

	Bangladesh rural						Bangladesh urban						Cambodia						China						Sri Lanka					
	v. violent	VAW	sex seeking	criminal without IPV/sex	least violent	*P* value	v. violent	VAW	sex seeking	criminal without IPV/sex	least violent	*P* value	v. violent	VAW	sex seeking	criminal without IPV/sex	least violent	*P* value	v. violent	VAW	sex seeking	criminal without IPV/sex	least violent	*P* value	v. violent	VAW	sex seeking	criminal without IPV/sex	least violent	*P* value
	1	2	3	4	5		1	2	3	4	5		1	2	3	4	5		1	2	3	4	5		1	2	3	4	5	
**Social characteristics**																														
Age group (years):																														
18-24	19.35	8.87	25.81	54.55	28.19	<0.0001	21.88	13.96	36.14	50	33.85	<0.0001	22.61	11.84	18.18	43.48	33.54	<0.0001	3.08	7.91	13.46	33.33	14.36	0.0002	14.93	13.98	12.9	38.18	39.21	<0.0001
25-34	22.58	25	45.16	18.18	28.95		43.75	33.78	46.99	40	35.42		36.52	39.47	52.27	34.78	34.67		35.38	22.6	38.46	42.86	28.37		47.76	34.41	43.55	35.45	31.17	
35-49	58.06	66.13	29.03	27.27	42.86		34.38	52.25	16.87	10	30.73		40.87	48.68	29.55	21.74	31.79		61.54	69.49	48.08	23.81	57.27		37.31	51.61	43.55	26.36	29.63	
Any high school	32.26	27.02	53.23	81.82	52.09	<0.0001	75	54.05	65.06	90	76.09	<0.0001	35.65	39.69	55.45	58.7	53.33	<0.0001	87.5	81.36	89.74	90.48	85.64	0.236	100	86.56	87.1	94.55	90.61	0.04
Ever married or cohabited	80.65	95.16	72.58	36.36	64.1	<0.0001	65.63	86.04	46.99	40	51.96	<0.0001	80	91.01	73.18	56.52	66.56	<0.0001	95.38	96.61	91.67	85.71	88.39	0.01	80.6	87.1	74.19	49.09	49.73	<0.0001
Current food insecurity	61.29	54.51	36.07	18.18	39.72	0.0002	53.13	39.82	32.93	30	24.55	<0.0001	66.07	61.9	45.58	41.3	47.22	<0.0001	30.16	24.56	20.13	19.05	19.26	0.291	29.23	17.95	27.12	18.18	14.9	0.003
Victimisation history:																														
Mother was abused	51.61	30.24	41.94	45.45	17.45	<0.0001	56.25	47.3	39.76	35	26.48	<0.0001	46.96	25.66	24.66	23.91	20.21	<0.0001	49.23	32.95	19.87	33.33	14.11	<0.0001	56.72	41.67	54.84	50	24.06	<0.0001
Fathers absence	51.61	50.83	45.16	40	46.13	0.682	59.38	52.25	42.17	60	45.78	0.149	36.84	43.52	42.92	39.13	38.54	0.352	12.5	19.21	11.54	14.29	13.07	0.245	29.85	37.63	41.94	38.18	33.39	0.45
Father contribution to housework:																														
Low	76.67	78.6	70.49	70	79.21	0.83	50	59.91	63.86	50	66.11	0.356	32.17	39.34	40.55	36.96	39.47	0.774	28.57	19.89	20	14.29	18.42	0.432	44.78	36.96	32.79	37.61	31.53	0.135
Mid	23.33	20.99	29.51	30	20.41		46.88	38.74	34.94	50	32.44		60.87	54.29	51.15	56.52	52.52		65.08	71.02	70.97	80.95	69.3		46.27	51.63	62.3	51.38	60.22	
High	0	0.41	0	0	0.38		3.13	1.35	1.2	0	1.45		6.96	6.37	8.29	6.52	8.02		6.35	9.09	9.03	4.76	12.28		8.96	11.41	4.92	11.01	8.25	
Childhood sexual abuse	51.61	35.48	27.42	36.36	15.3	<0.0001	56.25	55.86	46.99	55	30.06	<0.0001	28.7	18.64	18.26	13.04	10.77	<0.0001	36.92	16.57	16.67	14.29	6.96	<0.0001	36.36	23.03	25.81	22.73	9.93	<0.0001
Childhood physical abuse	29.03	22.58	3.23	36.36	9.73	<0.0001	46.88	28.38	28.92	50	14.53	<0.0001	53.04	53.95	51.6	56.52	37.74	<0.0001	56.92	38.07	34.62	28.57	16.15	<0.0001	69.7	55.62	56.45	61.82	29.87	<0.0001
Childhood emotional abuse or neglect	54.84	53.23	50	63.64	27.94	<0.0001	75	65.77	60.24	60	34.64	<0.0001	59.13	39.47	41.55	39.13	25.44	<0.0001	66.15	46.86	30.77	42.86	12.7	<0.0001	62.12	34.27	48.39	50	15.94	<0.0001
Teased or bullied as a child	32.3	32.7	25.8	18.2	21.5	0.038	50	44.14	38.55	45	23.91	<0.0001	48.7	39.65	43.18	39.13	30.36	<0.0001	53.85	35.23	30.13	19.05	17.16	<0.0001	37.88	25.14	27.42	38.18	13.75	<0.0001
Experienced homophobic abuse	29.03	8.61	6.56	18.18	3.7	<0.0001	31.25	3.17	7.32	5	4.17	<0.0001	21.24	1.35	2.33	6.52	1.37	<0.0001	19.35	7.06	5.23	0	3.4	0.0001	18.46	2.55	6.78	15.74	2.51	<0.0001
**Psychological factors & substance abuse**																														
Life satisfaction (mean)	0.19	0.10	-0.08	0.00	-0.12		0.37	0.25	0.37	0.30	-0.04		0.10	0.00	0.03	0.01	-0.02		0.67	0.16	-0.10	0.26	-0.11		0.47	0.13	0.06	0.12	-0.07	
Depression	61.29	48.79	45.16	63.64	35.9	0.0004	56.25	44.14	53.01	45	35.31	0.0054	62.61	56.42	45	45.65	33.85	<0.0001	55.38	38.15	33.55	38.1	18.58	<0.0001	23.08	25.64	26.67	20.37	10.38	<0.0001
Alcohol problems	0	0	0	0	0		15.63	3.6	6.02	15	0.78	<0.0001	53.91	34.65	42.73	45.65	19.08	<0.0001	33.85	14.12	20.51	14.29	11.42	<0.0001	56.72	22.58	29.03	37.27	12.36	<0.0001
**Gender attitudes and division of work**																														
Gender equity score: low	32.26	35.89	38.71	36.36	19.62	<0.0001	18.75	22.07	21.69	5	10.84	0.0022	26.96	31.36	18.18	13.04	16.72	<0.0001	3.13	2.26	4.55	4.76	1.42	0.142	23.88	17.22	26.23	6.73	11.42	0.0003
Man dominates some or all of decisions at home	68.18	54.24	48.89	25	53.28	0.442	57.14	50.26	48.72	50	40.17	0.109	45.45	46.38	46.9	44	40.87	0.337	51.61	41.86	42.66	42.11	22.55	<0.0001	51.85	43.95	65.91	28.85	25.36	<0.0001
Relationship control:																														
Low	29.03	40.32	32.26	27.27	31.98	<0.0001	18.75	38.29	20.48	45	32.18	<0.0001	26.96	42.98	39.09	32.61	39.38	<0.0001	1.54	5.08	4.49	9.52	5.19	0.0016	67.16	60.22	53.23	60	49.82	<0.0001
Mid	25.81	34.27	24.19	0	22.5		28.13	24.32	16.87	0	14.19		28.7	25	24.55	23.91	21.03		13.85	13.56	9.62	23.81	18.69		4.48	5.91	14.52	8.18	4.06	
Highly controlling	25.81	19.76	19.35	9.09	8.85		18.75	24.32	15.66	0	6.15		26.09	23.9	19.09	13.04	12.31		80	80.23	82.05	66.67	66.78		17.91	15.05	17.74	8.18	5.51	
Missing/no partner	19.35	5.65	24.19	63.64	36.66		34.38	13.06	46.99	55	47.49		18.26	8.11	17.27	30.43	27.28		4.62	1.13	3.85	0	9.34		10.45	18.82	14.52	23.64	40.61	

v. – very, VAW – violence against women

**Table 5:** Prevalence (%) of possible associated factors by masculinity class, Bougainville and Indonesia

	Papau New Guinea																	Indonesia Jakarta																	Indonesia Java rural																						Indonesia - Papua											
	V violent		VAW			Sex seeking				Criminal without IPV/sex		Least violent			P value			V violent			VAW			Sex seeking		Criminal without IPV/sex			Least violent			P value			V violent				VAW			Sex seeking			Criminal without ipv/sex			least violent			P value			V violent			VAW			Sex seeking			Criminal without IPV/sex			Least violent		P value
Social characteristics	1		2			3				4		5						1			2			3		4			5						1				2			3			4			5						1			2			3			4			5		
Age group (years):																																																																				
18-24	20.2		17.5			44.1				42.1		44.4			<0.0001			31.3			21.6			22.9		52.9			26.7			0.01			25.0				17.5			19.4			25.0			14.2			0.159			35.4			33.9			26.5			57.1			34.5		0.196
25-34	41.6		32.4			38.2				24.6		26.0						43.8			39.8			27.1		29.4			35.3						40.0				36.4			38.7			25.0			25.9						36.7			30.8			39.2			20.0			30.7		
35-49	38.2		50.2			17.7				33.3		29.6						25.0			38.6			50.0		17.7			38.0						35.0				46.1			41.9			50.0			59.8						28.0			35.2			34.3			22.9			34.8		
Any high school	40.6		45.4			35.3				47.4		43.4			0.598			87.5			79.5			77.1		88.2			81.1			0.424			95.0				80.5			71.0			90.0			69.8			0.009			90.7			91.6			91.2			100.0			92.7		0.340
Ever married or cohabited	86.3		90.5			73.5				54.4		51.5			<0.0001			64.6			77.8			72.9		37.3			64.9			0.002			65.0				79.2			87.1			70.0			80.3			0.299			70.2			69.6			62.8			48.6			59.2		0.03
Current food insecurity	58.0		39.1			60.6				35.1		33.3			<0.0001			25.5			9.6			12.8		19.6			9.9			0.01			15.8				10.6			29.0			10.0			8.3			0.009			28.5			17.6			19.6			25.7			9.7		0.0009
Victimisation history:																																																																				
Mother was abused	66.8		56.9			47.1				56.1		42.6			0.002			25.0			12.9			14.6		5.9			6.6			0.004				30.0					13.6			16.1		15.0			5.4			0.009			41.0			31.9			30.4			25.7		11.5		<0.0001
Father away most or all the time	48.1		30.2			41.2				35.1		25.9			0.0001			56.3			37.4			35.4		39.2			28.9			0.025				30.0					29.9			25.8		30.0			19.2			0.104			36.0			32.2			36.3			25.7		28.3		0.181
Father contribution to housework:																																																																				
Low	17.6		15.0			20.6				22.8		16.4			0.344			60.4			53.9			45.8		51.0			46.4			0.367				50.0					39.2			48.4		50.0			32.7			0.218			21.9			21.2			20.6			20.0		26.6		0.158
Mid	67.2		66.6			76.5				68.4		68.7						35.4			42.0			43.8		45.1			46.0							50.0					54.3			38.7		40.0			59.0						54.4			61.5			53.9			48.6		58.7		
High	15.3		18.5			2.9				8.8		14.9						4.2			4.1			10.4		3.9			7.7							0.0					6.5			12.9		10.0			8.3						23.7			17.3			25.5			31.4		14.7		
Childhood sexual abuse	48.5		28.4			32.4				26.3		15.9			<0.0001			35.4			8.2			4.2		5.9			4.2			<0.0001				15.0					13.6			9.7		15.0			3.6			0.0005			20.5			14.2			11.8			20.0		4.9		0.0001
Childhood physical abuse	78.6		66.8			76.5				57.9		54.9			<0.0001			43.8			45.3			27.1		51.0			27.1			0.0003				45.0					29.9			16.1		55.0			13.1			0.0007			71.43			58.4			54.9			62.9		33.6		<0.0001
Childhood emotional abuse or neglect	73.3		59.7			55.9				49.1		41.5			<0.0001			47.9			32.4			18.8		29.4			17.1			0.0002				35.0					24.7			22.6		20.0			9.9			0.0004			55.28			49.6			37.3			45.7		18.0		<0.0001
Teased or bullied as a child	67.1		57.5			66.7				71.9		45.6			0.0002			39.6			35.9			27.1		29.4			19.5			0.0003				30.0					28.6			19.4		30.0			15.3			0.004			34.78			35.8			29.4			37.1		16.7		0.0003
Experienced homophobic abuse	14.0		3.3			6.1				5.3		2.1			<0.0001			4.3			0.0			2.1		0.0			0.4			0.0148				5.3					1.3			0.0		0.0			0.4			0.173			1.89			1.4			2.0			0.0		0.3		0.578
Psychological factors & substance abuse:																																																																				
Life satisfaction (mean) (low score = higher satisfaction)		0.16			-0.12			-0.05			-0.06			0.01						0.53			0.38	0.20				0.31			0.09							-0.02			0.15			-0.16		0.48			0.02						-0.03			-0.30			-0.29			-0.50		-0.23		
Depression (high = more depressed)		54.2			42.4			50.0			42.1			25.6			<0.0001			45.8			25.9	25.0				29.4			15.3			0.0007				40.0			32.5			25.8		15.0			10.4			<0.0001			34.2			25.8			24.5			22.9		19.2		0.029
Alcohol problems		69			43			65			58			34.2			<0.0001			52.1			11.1	22.9				49.0			7.1			<0.0001				45.0			7.8			16.1		15.0			1.7			<0.0001			72.7			28.2			49.0			51.4		10.9		<0.0001
Gender attitudes and division of work:																																																																				
Gender equity score: low				22			22		41		33		18.4			0.116			16.7			4.1			4.2		7.8			4.7			0.038				10.0			5.3			6.5		0.0		3.8			0.409			20.8			23.0			15.7			11.4			17.5		0.272	
Man dominates some or all of decisions at home				45			37		28		48		35.7			0.219			58.1			49.6			60.6		68.4			43.7			0.020				61.5			32.2			48.2		35.7		37.4			0.253			34.0			27.3			21.7			23.5			16.8		0.056	
Relationship control:																																																																				
Low				42			50		32		51		38.3			<0.0001			8.3			20.5			18.8		15.7			25.1			0.0227				5.0			16.2			12.9		10.0		22.7			0.283			9.9			15.0			18.6			8.6			18.8		0.073	
Mid				18			17		12		4		11.2						37.5			31.0			37.5		45.1			30.4							35.0			40.9			32.3		40.0		33.1						24.2			22.0			35.3			28.6			23.4			
highly controlling				29			26		41		16		11.2						45.8			43.9			41.7		37.3			34.2							55.0			34.4			51.6		40.0		33.7						61.5			59.5			42.2			62.9			50.5			
missing/no partner				10			7		15		30		39.3						8.3			4.7			2.1		2.0			10.4							5.0			8.4			3.2		10.0		10.5						4.4			3.5			3.9			0.0			7.3			

CI – confidence interval, VAW – violence against women

In five of the nine settings high school attendance was associated with class. Most commonly the highest high school attendance was associated with being in the community violence class (Class 4). However, in most settings Class 1 membership was associated with very high proportions of men having attended high school, and in rural Indonesia and Sri Lanka high school attendance was the highest in this class. Notable exceptions were found in rural Bangladesh and Cambodia where high school attendance was particularly low in Class 1. Marital status was associated with class membership in all settings except rural Java, but the most conspicuous pattern was of lower marriage or cohabiting rates associated with Class 4.

Current food insecurity was associated with class membership in all settings except China. In all settings food insecurity was the lowest, or the proportion close to that of the lowest class, for men in Class 5 (lowest violence). In eight of the nine settings food insecurity was the highest (or approximately joint highest) among men in Class 1 (highest violence), the exception was rural Java where Class 3 had the highest food insecurity.

In all settings having experienced sexual, physical or emotional abuse or neglect in childhood was associated with Class membership. All of these forms of abuse (or neglect) experience were lowest among men in Class 5. In every setting childhood abuse experience was most common for men in Class 1, with the exception of rural Bangladesh where childhood physical abuse and emotional abuse were highest for Class 4 men. The same pattern was visible in men’s reports of having witnessed abuse of their mothers by their father, this was in every setting highest in Class 1 and lowest in Class 5. Other behaviour of the men’s father was mostly not associated with class membership. There was no association between class and his contribution to domestic work. In only two settings was father’s absence in childhood associated with men’s class. These were Bougainville and Indonesia Jakarta. In both of these settings, father’s absence was highest for men in Class 1 and lowest for those in Class 5.

Life satisfaction levels were patterned by masculinity Class in all settings except Cambodia and rural Bangladesh. In all the other countries the life satisfaction of Class 1 men was the lowest, and was significantly different from that of Class 5 men in urban Bangladesh, Jakarta, China and Sri Lanka. The life satisfaction of Class 1 men was significantly lower than those of Class 2 men in Indonesia Papua, China, Sri Lanka and Bougainville. It was significantly lower than Class 3 men’s satisfaction in China and Sri Lanka, and significantly lower than that of Class 4 men in Indonesia Papua and Sri Lanka (*P* values not shown).

In all settings depression and problem drinking was associated with Class, with the highest levels found among men in Class 1 and the lowest in Class 5. The situation with problem drinking was the same. Men in the most violent class (Class1) had the highest prevalence of problem drinking and men in the least violent had the lowest (Class 5).

The proportion of men expressing the most gender inequitable attitudes was significantly different across classes in urban and rural Bangladesh, Cambodia, Sri Lanka and Indonesia Jakarta. The proportion of men with most gender inequitable attitudes was the lowest in Class 5 in many of the settings, but in Cambodia it was lowest of all among Class 4 men. Class 1 men had a high proportion in all settings. Only in Cambodia, China and Sri Lanka were there differences between Classes in men’s dominance of decision-making. This was reported to be most equitable among Class 5 men.

**Table 6** shows the findings of the multinomial logistic regression model of factors associated with class membership in the pooled data. Comparing Class 1 and Class 5 men, those in Class 1 were more food insecure, more likely to engage in problem drinking, had lower life satisfaction and were more depressed. They dominated household decision-making more and were more likely to be very controlling of their partner. Class 2 men were very similar in these respects to those of Class 1, the only difference being that they were also more likely to hold very gender inequitable attitudes compared to Class 5 men. Comparing Class 3 to Class 5, Class 3 men were more likely to engage in problem drinking and be depressed, to have very gender inequitable attitudes, dominate many household decisions and to be very highly controlling of their wife or girlfriend. There was also some evidence that they were somewhat older than the men in Class 5, with more of them in the 25-34 years age group than in the 18-24 group. Comparing Class 4 and Class 5 men, Class 4 men were younger (more were 18-24 years), and more likely to be depressed and to engage in problem drinking (**Table 6**).

**Table 6:** Multinomial regression model of social and attitudinal factors associated with masculinities classes (N = 9753), all countries adjusted for age and site

	1 vs 5			2 vs 5			3 vs 5			4 vs 5		
	**Relative Risk Ratio**	**95% CI**	**P value**	**Relative Risk Ratio**	**95%CI**	**P value**	**Relative Risk Ratio**	**95% CI**	**P value**	**Relative Risk Ratio**	**95% CI**	**P value**
Food insecurity	1.79	(1.44, 2.20)	<0.0001	1.24	(1.10, 1.41)	0.001	1.14	(0.95, 1.36)	0.157	1.02	(0.79, 1.32)	0.877
Problem drinking	11.06	(8.55, 14.32)	<0.0001	3.02	(2.46, 3.72)	<0.0001	3.95	(3.07, 5.06)	<0.0001	7.04	(5.14, 9.64)	<0.0001
Life satisfaction (high score = poor satisfaction)	1.34	(1.22, 1.46)	<0.0001	1.17	(1.10, 1.24)	<0.0001	1.05	(0.97, 1.14)	0.191	1.09	(0.98, 1.21)	0.118
Depressed	2.52	(2.03, 3.13)	<0.0001	1.92	(1.70, 2.18)	<0.0001	1.70	(1.45, 1.99)	<0.0001	1.61	(1.25, 2.08)	<0.0001
Highly gender inequitable	1.33	(1.03, 1.73)	0.03	1.55	(1.30, 1.84)	<0.0001	1.46	(1.14, 1.85)	0.002	1.06	(0.76, 1.48)	0.745
Man dominates some or all of decisions at home	1.78	(1.45, 2.18)	<0.0001	1.32	(1.16, 1.50)	<0.0001	1.46	(1.19, 1.77)	<0.0001	1.27	(0.95, 1.71)	0.108
Relationship control: low	1.00			1.00			1.00					
Mid	1.43	(1.06, 1.90)	0.014	1.13	(0.97, 1.32)	0.122	1.19	(0.92, 1.53)	0.175	0.93	(0.66, 1.32)	0.348
Highly controlling	1.90	(1.45, 2.48)	<0.0001	1.44	(1.24, 1.66)	<0.0001	1.46	(1.16, 1.84)	0.001	0.97	(0.71, 1.32)	0.295
Age groups (years):												
18-24	1.00			1.00			1.00					
25-34	1.44	(1.11, 1.88)	0.835	1.09	(0.89, 1.33)	0.41	1.79	(1.40, 2.30)	<0.0001	0.71	(0.52, 0.98)	0.04
35-49	1.09	(0.80, 1.47)	0.384	1.12	(0.90, 1.39)	0.324	1.12	(0.83, 1.53)	0.455	0.54	(0.37, 0.78)	0.001

CI – confidence interval

Antecedent factors associated with Class memberships are shown in Table 7 in multinomial logistic regressions. Men in Classes 1 and 2, compared to Class 5 were more likely to have witnessed their mother being abused. There was no difference in the relative risk of men having experienced their father never or rarely being at home when they were growing up, or in their father participating in domestic work in any of the other Classes, compared to Class 5. There was a significantly higher relative risk of having experienced physical abuse and emotional abuse and neglect were among men of all Classes compared to those in Class 5. There was a similar finding for sexual abuse for Classes 1, 2, and 3. The men of all classes, compared to class 5 had a higher relative risk of having been teased or bullied as children. Men of Classes 1 and 4, compared to Class 5, were much more likely to have reported ever having been called by names or faced derogatory remarks or been subjected to threats of violence or actual violence because they were thought to be effeminate or ‘sissy’ (homophobic abuse or violence). The men in Class 2, when compared to Class 5, were less likely to have attended high school, and those in Class 4 were more likely to have done so (**Table 7**)

**Table 7:** Multinomial regression model of childhood exposures associated with masculinities classes of ever partnered men (N = 9806), all countries adjusted for age and site

	1 vs 5			2 vs 5			3 vs 5			4 vs 5		
	**Relative risk ratio**	**95% CI**	**P value**	**Relative risk ratio**	**95%CI**	**P value**	**Relative risk ratio**	**95% CI**	**P value**	**Relative risk ratio**	**95% CI**	**P value**
Mother was abused	1.85	(1.49, 2.30)	<0.0001	1.24	(1.07, 1.45)	0.005	1.23	(1.00, 1.52)	0.046	1.14	(0.88, 1.49)	0.325
Father rarely or never at home	1.19	(0.98, 1.45)	0.085	1.12	(0.98, 1.27)	0.09	1.06	(0.88, 1.26)	0.549	1.12	(0.88, 1.44)	0.354
Father contribution to housework: low	1.00			1.00			1.00			1.00		
Mid	0.95	(0.78, 1.16)	0.617	1.02	(0.90, 1.15)	0.797	0.98	(0.82, 1.17)	0.833	0.90	(0.70, 1.16)	0.43
High	1.08	(0.78, 1.48)	0.654	1.02	(0.82, 1.26)	0.88	1.10	(0.82, 1.48)	0.518	1.01	(0.66, 1.53)	0.971
Childhood sexual abuse	2.63	(2.06, 3.34)	<0.0001	1.71	(1.46, 2.00)	<0.0001	1.51	(1.18, 1.92)	0.001	1.32	(0.97, 1.81)	0.077
Childhood physical abuse	2.10	(1.71, 2.60)	<0.0001	1.75	(1.53, 2.00)	<0.0001	1.53	(1.29, 1.82)	<0.0001	2.09	(1.63, 2.69)	<0.0001
Childhood emotional abuse or neglect	1.36	(1.26, 1.46)	<0.0001	1.25	(1.19, 1.31)	<0.0001	1.20	(1.12, 1.29)	<0.0001	1.21	(1.12, 1.32)	<0.0001
Was teased or bullied as a child	1.56	(1.28, 1.89)	<0.0001	1.57	(1.39, 1.78)	<0.0001	1.47	(1.24, 1.74)	<0.0001	1.67	(1.32, 2.12)	<0.0001
Experienced homophobic abuse	7.35	(5.08, 10.65)	<0.0001	1.37	(0.97, 1.93)	0.072	1.66	(1.07, 2.57)	0.023	2.70	(1.66, 4.39)	<0.0001
Any high school	0.97	(0.80, 1.17)	0.752	0.79	(0.69, 0.90)	0.001	1.00	(0.82, 1.24)	0.945	1.51	(1.12, 2.03)	0.007

CI – confidence interval

## DISCUSSION

We have shown that across the nine sites in six countries men could be grouped into a set of classes according to their probability of having engaged in different configurations of acts of violence against women, anti-social or illegal behaviour and sexually risky or predatory practices. The five groups (classes) provide some insight into the structure of masculinities within the Asia-Pacific region and had many similarities with the results of a similar latent class analysis undertaken in a population-based sample of men in South Africa. Our analysis suggests that globally there is a consistent patterning of masculinities in relation to violence and the exercise of sexual and economic power over women, and this fits an argument that there is a global gender regime that structures men’s practices [14,16], rather than local ‘culture’. It does not mean that more violent masculinities are hegemonic, indeed they are probably not, but it does suggest that violence has a central position in structuring masculinities.

In our findings, as in the population-based samples from South Africa and one from the USA [15,23,32], the largest class of men was the least violent one. In the Asia-Pacific region 59.5% of men were allocated to this, compared to 45.7% in South Africa. This shows that most commonly men are not very physically or sexually violent, although this does not equate with being gender equitable and these men still benefitted from patriarchal privilege and many held patriarchal ideas and values and had used emotional or economic power over their partners. The second largest class in Asia-Pacific (21.2%) was similarly generally law abiding, but the men in this class had been very violent towards women partners and were highly gender inequitable and controlling. This class had many similarities with the second largest group of men in the population-based studies in South Africa [15]. In Asia-Pacific there was a third class (7.8% of men) where allocated men were notably generally law abiding (ie, few had been in gangs, fought with weapons or used drugs), but they were positioned most notably at the nexus of sex and power. Many had been violent towards women, but less markedly so than in Class 2, but a defining feature of the class was the propensity of these men to have had transactional sex, sex with sex workers and multiple sexual partners in the past year. Many of them had raped. This has similarities with a class identified in the USA [32]. There were two classes in Asia and the Pacific where men were notably engaged in anti-social behaviour. One of these was a class (7.9% of men allocated) that was very similar to the most highly violent men in all three studies in South Africa, and to Logan-Greene and Davis’s most violent men [33], corresponding to the descriptions of hyper-masculinity of Herek and colleagues [34,35]. In this group, men had a very high probability of having been involved in all anti-social behaviours, acts of violence against women and acts at the nexus of sex and power. The community violence class was the smallest (3.6%), and had similarities with class 1, except that the men had a much lower probability of having been physically or sexually violent towards women and few had had sex with sex workers. A key feature of this class of men is that more of them were younger than those in the other classes. Giving rise to the possibility that without intervention they would grow increasingly similar to class 1 men.

There is an ongoing debate about the extent to which there exists a global configuration of masculinity(ies) or there is significant variation shaped by local contexts [14,36,37]. While there was variation between the proportions of men allocated to each class by site, for instance in the highest violence class, less than 3% of men in both Bangladesh sites and rural Java were allocated to it, compared to 30.3% in Bougainville, the five classes did occur in all sites and were important for understanding masculinity positions. However, the least violent masculinity was not the most common in all settings and in Bougainville, the category of law abiding but very violent towards women (class 2) was the most common, and the generally violent and engaged in anti-social activities (class 1) was the second most common. The least violent men in Bougainville only constituted 22.7% overall. Bougainville was different from the other settings in that it had recently emerged from a conflict which had engulfed the whole island. It seems likely that this was related to the much higher expression of more violent masculinities in the setting [38,39]. A similar dynamic may be at play in Indonesia Papua, which has been marked by over five decades of low-level insurgency and high levels of militarization.

We have shown that class membership is associated with poverty, men’s worse psychological state and patriarchal power. The men in the classes displaying most violence towards women were the most likely to report current food-insecurity, and they also had the poorest life satisfaction. This provides some quantitative support for the arguments made from ethnographic research of Phillipe Bourgois, and reflected by other authors, that in contexts of poverty and when men feel unable to occupy positions as successful men they direct their more energies to asserting power and control over women [20,40,41]. It also is supported by analyses from variated settings linking men’s poverty and perpetration of IPV [1,10,26]. For all classes, compared to the least violent men, there was an association with more hazardous alcohol use and depression. This supports contentions that having more conflictual relations of dominance over women and other men does not make men very happy or healthy [27].

There were three indicators of gender inequity: men’s attitudes, dominance of decisions at home and control of their wife or girlfriend. Men who were very violent towards women, and in the class at the nexus of sex and power were very similarly positioned in respect of these. The men in the most violent class were the most dominant in their households and controlling. This analysis supports the central position of gender power and relations in understanding men’s relations with other men, and engagement in anti-social practices as well as in understanding men’s violence against women. In four settings however, China, Bougainville, Indonesia (rural Java), Indonesia (Papua), gender attitudes were not descriptively associated with the different classes, but in these settings expressed attitudes correlated poorly with men’s dominance in decision-making and/or relationship control. For example, in China, whilst gender attitudes were not very inequitable, men’s practices were very highly controlling and patterned by masculinity Class membership. This points to a level of disconnect between how men express ideas of gender relations and what they expect in practice.

The analysis of childhood and trauma exposure variables provided an opportunity to examine associations with a range of variables to which exposure would, very likely, have preceded engagement in behaviours that led to class assignment. The associations between childhood trauma exposure, being teased, experiencing homophobic abuse and class assignment echo the findings of the analysis on data from South Africa [15,22], as well as analysis of factors associated with violence perpetration from multiple analyses [10,11,13,14]. This strongly supports arguments that men’s adult practices are highly influenced by their experiences in childhood [28,42,43]. Understanding that gender-based and other criminal violence has a life course development trajectory is critical for public sector policy and development work.

High school education was differently patterned across two of the groups in comparison to class 5, with class 2 men who were very violent towards women being less likely to have high school education, and men in the community violence class (class 4) being more likely . These findings echo the literature which shows a number of studies have found education may reduce men’s perpetration of violence, through supporting more gender equitable attitudes, but can increase their sense of entitlement and in this context has been sometimes shown associated with a greater risk of having raped [14,22].

One might expect that the masculinity men adopt may have been shaped by their father’s, and their father’s practices. This was true, in our analysis, in relation to men who witnessed their mothers being abused as they more likely to themselves be allocated to classes marked by violence. This is a very well recognised finding [11]. We also explored whether more positive behaviour of the men’s father’s – participation in domestic work – might have influenced their class assignment, and we showed that it did not. It rather appears that it was the traumatic exposures in childhood that had a greater impact on shaping how boys transitioned into men.

The assignment of men into classes based on patterning of behaviours provides an opportunity to reflect on patterning of different forms of violence against women and men’s other violent and anti-social practices, as well as practices at the nexus of sex and power. The most violent class shows us that in the case of some men, all of these practices are very highly correlated. However, for the Class 2 men, who are very violent towards partners, we see these violent practices correlated with transactional sex, but not correlated with non-partner rape perpetration. Research on non-partner sexual violence from the global south, has tended to emphasise its overlaps with IPV [12,13], but in our analysis we see it correlated with the other violent and anti-social practices, including gang membership, but not necessarily with IPV. The only other class where non-partner rape was a defining feature of class assignment was the class positioned at the nexus of sex and power. Here rape perpetration is strongly correlated with men having multiple partners, and using material power to gain sexual access to women. This does suggest that there may be a second group of men who rape who perceive a need to demonstrate sexual power over women. This finding may also reflect the small group of men who were markedly orientated towards sex in Logan-Greene and Davis’s study [33]. This finding also resonates with those from South Africa, and provides a link between a class of masculinity and sexually risky behaviours and underscores the argument that part of the efforts to promote safer sexual practices should involve changing masculinities rather than just focusing on changing individual male behaviours [8].

This identification of two classes of men who rape is highly resonant of Malamuth et al’s Confluence model that was developed to explain rape perpetration among college students in the USA [29], although it goes considerably beyond this in studying engagement in anti-social behaviour, which was more in keeping with the emphasis placed by Knight and Sims-Knight [30]. The model has two pathways, the first is a hostile masculinity pathway, where men express cynical, adversarial views on relations with women and with a strong correlation to general acceptance of the use of interpersonal violence, and a second pathway in the confluence model that emphasizes heterosexual performance. It has very important implications for non-partner rape prevention, as it suggests that a dual strategy for prevention may be needed. The first element are interventions to prevent men’s engagement in gangs and general criminal activities, which are often homosocial in that they are conducted with other men [44,45]. The second element are interventions to address the psychological basis of the perceived need to demonstrate sexual power over women. The origins of this second group need to be better understood, but we have shown that they are highly patriarchal and have substantial substance abuse and psychological problems, in part stemming from a notable background of abuse experience. This is very strongly in keeping with Malamuth et al’s explanation for his second pathway [29].

Masculinities are fluid and dynamic, as well as multiple. Our presentation of five classes of masculinity was the model that best fitted our data, but had the data set been larger we may have identified other classes of masculinities that are less common within the general population. We were also restricted to the available variables for definition of classes and possibly having others would have altered some men’s class assignment. We would also not like the analysis to be interpreted as indicating that masculinities are fixed, although for this analysis we have fixed them in categories based on their reported practices. Some of the variables used were lifetime rather than past year exposures and had the former been available in longitudinal analysis we may have seen change over time and so individual men might move between masculinity categories.

A key question is whether any of the masculinity classes would be considered as socially most powerful or ‘hegemonic’, and indeed whether analysis such as this can identify it. The term ‘hegemonic masculinity’ has been used in masculinity studies to variously refer to social and political mechanisms to ensure consent to the continuation of patriarchy, to the most powerful, celebrated and/or most widespread versions of manhood at a given time in a given society, or to refer to specific groups of men [46]. While common usage among gender activists and some policy makers, often equates hegemonic masculinity with violent or ‘toxic’ masculinities, this has been questioned both from a theoretical and an empirical standpoint [47,48]. Male perpetration of IPV may be often tolerated up to a point or normalized in society, but it is seldom celebrated [49]. In the sample here, the numerically most dominant category was the least violent and most law-abiding, but we would caution against assuming that this was viewed within the communities studied as necessarily the most admired and powerful position. Indeed our data does not provide information on which class of masculinity was hegemonic, and this is likely mediated through other factors such as local social and legal norms and may differ by social class, age groups and community. Furthermore, research from other settings has highlighted the uneven relationship between social power and numerical dominance [36,46,50]. However, the knowledge that the least violent group is the most numerically large may be very important for practitioners working to transform gender norms as it provides an entry point for discussions even if it is not recognized as the most socially revered position. Certainly shifting other masculinities towards this, as well as building gender equality throughout masculine positions, would be the goals of such work.

Whilst authors describe hypermasculinity as a separate form of masculinity, they also view it as an intensification of hegemonic masculinity, rather than as completely distinct. Our analysis suggests that the most violent group (Class 1) has many behavioural overlaps with Classes 2-4. In the analysis from South Africa there was considerable confidence that men who were violent to their partners, but otherwise not hugely anti-social reflected the hegemonic masculinity based on the substantial body of qualitative work describing this [15,19]. In our data set we draw data from very varied settings and lack the confidence to make a pronouncement about which position may be considered hegemonic, and would not to be so bold as to suggest that there is one hegemonic masculinity in Asia and the Pacific, given that its diverse population makes up more than half of humankind, even if we can apportion men based on their responses to our questionnaire at the time of interview to a limited number of classes.

### Strengths and limitations

This analysis was based on a large data set from a multi-country study, that was conducted in a manner that ensured that the data collected from different sites was comparable. The sites were diverse and so the findings are by implication generalizable. However, in the analysis it was assumed that the classification of the classes was done without error. The uncertainty, indicated by quite a mediocre entropy value of 0.65, was not taken into account in further regression analysis, which could affect the standard errors of the regression results. To cover this limitation to some extent, we have interpreted only predictors with a *P* value of ≤0.01 as statistically significantly associated in the final multiple logistic regression model. Further, it was shown that the proportion of men across the five classes for Bougainville was different to the proportion of men across the five classes for other countries. Future research might explore whether there would be a difference in indicators that determine the classes for Bougainville, if the data were analysed on their own, and whether the optimal model is equivalent regarding the number of classes compared to the other countries.

This analysis was conducted on available data from the UN Multi-country Study and on Men and Violence in Asia and the Pacific and its findings may not be generalizable outside the contexts in which the data were collected. It is possible that if other countries or sites within countries in this region were included that the findings would be different. There may be error in men’s reports of their lives, violence perpetration and experiences in childhood, including concealed memory of childhood trauma and distorted perceptions of parental engagement at home.

## CONCLUSIONS

In this paper we have shown that in a very large multi-country data set from Asia and the Pacific, men’s probabilities of engagement in a range of practices at the nexus of gendered power and violence, including anti-social practices, follow a pattern that this can be used to differentiate men into different classes. This provides a quantitative base for understanding both the diversity of men’s practices as well as operationalizing the notion in masculinity theory of multiple masculinities. We have shown the childhood origins of many of these classes, the links to poverty and a strong association with depression and low life satisfaction and men’s use of violence which points to men externalizing their own psychological distress and dissatisfaction towards their women partners in an expression of their patriarchal power.

A central challenge in prevention of IPV and other violence against women is gender transformative programming, ie, changing the way men see and express themselves as men. In class terms, that is transitioning men from Classes 1, 2, 3 and 4 towards Class 5. Our analysis has highlighted the importance of viewing men’s practices as grouped and driven centrally by an ideological framework which may be expressed somewhat differently and have different social and religious underpinnings in different contexts, but fundamentally translates men’s patriarchal power into a range of practices which have different relations to the subjugation of women and competition among men. These practices transcend settings, to the extent that they are found to different degrees, rather similarly grouped in very diverse cultural and social contexts. Understood in this way, it is the challenge of changing the way men see themselves as men and the practices which flow from this that is the central challenge of gender transformative practice. It may be, however, that different strategies are need for working with different classes of men, as seen in initial research. To the extent that we have demonstrated again connections with poverty, conflict, lower levels of schooling and psychological factors, including substance abuse, in the longer term addressing these must be part of the programme for changing men. Gender transformative programming may be more effective if it acknowledges the substantial psychological distress of many of the intended men, as well as their needs for interventions to assist in poverty reduction.

## Additional material

Online Supplementary Document
