# Supplementary material for: Masculinities and violence: using latent class analysis to investigate the origins and correlates of differences between men in the cross-sectional UN Multi-country Study on men and violence in Asia and the Pacific
Source: J Glob Health. 2020 Dec 19;10(2):020439. doi: 10.7189/jogh.10.020439 (PMC7774029; doi:10.7189/jogh.10.020439)
Supplement: Online Supplementary Document [file jogh-10-020439-s001.pdf]

Table S1 Model fit criteria

classes	cov	# free parameters	Log likelihood	AIC	BIC	adjBIC	entropy	Vuong-Lo_Menndell-Rubin LRT for k vs k-1 classes (LMR LR)	*P* value	Lo-Mendell-Rubin adjusted LRT (ALMR LR)	*P* value	Bootstrap LRT for k vs 2k-1 classes (BLRT)	*P* value *
2	no	21	-39324	78689	78840	78774	0.741	-42406	0.000	6104	0.000	-42406	0.000
3	no	32	-38844	77751	77983	77881	0.613	-39324	0.000	950	0.000	-39324	0.000
4	no	43	-38594	77274	77585	7749	0.595	-38844	0.000	494	0.000	-38844	0.000
5	no	54	-38416	76939	77330	77158	0.648	-38594	0.000	354	0.000	-38594	0.000
2	country 1	22	-39198	78439	78598	78528	0.749	-42406	0.000	6358	0.000	-42406	0.000
5†	country 1	58	-37977	76071	76490	76306	0.692	-38282	0.000	603	0.000	-38282	0.000

*a significant P value indicates a significant improvement in model fit in the k class model compared to the k-1 class model

†One or more multinomial logit parameters were fixed to avoid singularity of the information matrix. The singularity is most likely because the model is not identified, or because of empty cells in the joint distribution of the categorical latent variables and any independent variables. The following parameters were fixed: parameter 58, c#4 on country1
